# Clinical and Functional Effectiveness of Botulinum Toxin in Temporomandibular Disorders: A Systematic Review of Randomized Controlled Trials

**DOI:** 10.3390/toxins18100417

**Published:** 2026-09-28

**Authors:** Gianpaolo Ronconi, Rossella Calciano, Alberto Cutaia, Rosa Cioffi, Eslam Abdelkhalek Farag, Anna Sanfilippo, Martina Ferrillo, Alessandro de Sire, Paola Emilia Ferrara

**Affiliations:** 1 Department of Geriatrics, Orthopedics and Rheumatology, Fondazione Policlinico Universitario “Agostino Gemelli” IRCCS, Largo A. Gemelli 8, 00168 Rome, Italy; gianpaolo.ronconi@policlinicogemelli.it (G.R.); rossella.calciano@guest.policlinicogemelli.it (R.C.); paolaemilia.ferrara@policlinicogemelli.it (P.E.F.); 2 University Department of Geriatric and Orthopedic Sciences, Università Cattolica del Sacro Cuore, Largo Francesco Vito 1, 00168 Rome, Italy; alberto.cutaia@guest.policlinicogemelli.it (A.C.); dr.cioffirosa@gmail.com (R.C.); eslamfenoun2020@libero.it (E.A.F.); anna.sanfilippo@guest.policlinicogemelli.it (A.S.); 3 Dentistry Unit, Department of Health Sciences, University of Catanzaro “Magna Graecia”, 88100 Catanzaro, Italy; martina.ferrillo@unicz.it; 4 Department of Life Sciences, Health, and Health Professions, Link Campus University, Viale del Casale di S. Pio V, 44, 00165 Rome, Italy

**Keywords:** botulinum toxin, temporomandibular disorders, bruxism, orofacial pain, myofascial pain, conservative treatment

## Abstract

Temporomandibular disorders (TMDs) represent a heterogeneous group of conditions, including myogenous and arthrogenous, with a significant impact on quality of life. Botulinum toxin type A (BoNT-A) has been proposed as a therapeutic option, particularly for patients with refractory myogenous TMD and bruxism. This systematic review aimed to evaluate the clinical effectiveness of BoNT-A in the management of TMDs overall and bruxism, with particular focus on myogenous TMDs compared to placebo and non-surgical treatments. RCTs published between 2020 and 2025 were identified through PubMed, Scopus, and Google Scholar. Overall, 15 RCTs involving 797 participants were included. The available evidence suggests potential benefits of BoNT-A, particularly in patients with localized myofascial pain, myogenous TMD, or marked masticatory muscle hyperactivity. Interpretation of the findings is limited by substantial heterogeneity in treatment protocols, toxin dosage, diagnostic criteria, outcome measures, and follow-up duration. Current evidence does not support the routine use of BoNT-A as a first-line treatment for TMDs overall. Instead, BoNT-A may represent an adjunctive or second-line option for selected patients with myogenous TMD, localized myofascial pain, or marked masticatory muscle hyperactivity who do not adequately respond to conventional noninvasive treatment. Larger and methodologically rigorous RCTs are needed to establish appropriate indications, duration of treatment effects, and long-term safety across different TMD subtypes.

## 1. Introduction

Temporomandibular disorders (TMD) includes a series of pathological conditions affecting the masticatory muscles, the temporomandibular joint (TMJ) and related tissues. These conditions are among the main causes of non-odontogenic orofacial discomfort and can seriously compromise the patient’s quality of life. Movements such as mouth opening and closing, mastication, speech, and swallowing are all made possible by the temporomandibular joint, which connects the mandible to the temporal bone of the skull [1].

When this joint or the associated muscles experience functional or inflammatory alterations, the following symptoms appear: pain in the mandible and face, stiffness in the muscles, restrictions of mouth movements, headache, articular sounds (such as clicking or crepitus), and mastication difficulties [2]. TMD has multifactorial causes, such as psychological stress, bruxism, muscle overload, trauma, alterations of posture, and articular and neuromuscular factors [3].

TMDs are often recognized as complex biopsychosocial conditions, in which biological, psychological, and social factors interact to influence their severity. They may also present with other chronic pain disorders and disturbances in general health. The OPPERA studies highlighted that a greater number of comorbid pain conditions was associated with an increased risk of first-onset TMD, further supporting the view that TMD should not be considered solely as a localized orofacial pain condition [4,5].

Moreover, OPPERA findings underlined the contribution of psychological functioning, health status, pain sensitivity, and clinical orofacial factors to TMD development, supporting a multifactorial biopsychosocial model of TMD [6].

However, not all TMD-related findings are pathological or clinically symptomatic. Given the focus of this review on BoNT-A and its primary effects on muscle activity, the present discussion is specifically focused on myogenous TMD, particularly masticatory muscle pain (myalgia), rather than on TMDs as a whole [2,7].

Diagnostic assessment is based on the evaluation of specific clinical signs, including familiar pain elicited during mandibular movements or muscle palpation, restricted mouth opening, joint sounds, and positive responses to pain provocation tests. To enhance diagnostic reliability and reduce false-positive findings, the protocol specifically emphasizes the reproduction of the patient’s familiar pain, defined as pain consistent with the patient’s chief complaint [8]. The TMD Pain Screener, jaw function tests, pain drawings, and psychosocial scales are crucial because TMD patients’ perception of pain and the intensity of their symptoms are often influenced by behavioral and psychological factors [2].

Furthermore, diagnostic techniques, like electromyography (EMG) and polysomnography (PSG), are used in research studies examining temporomandibular disorders and sleep bruxism. PSG is still regarded as the gold standard for diagnosing sleep bruxism, even though EMG enables quantitative evaluation of masticatory muscle activity. However, the inconsistent results about the effectiveness of botulinum toxin can, at least partially, be due to variations in the diagnostic techniques used in different investigations. As regards treatments available, they may be divided into conservative and non-conservative ones. Rehabilitation strategies for TMDs are heterogeneous, and they often involve an integrated multidisciplinary approach combining physiotherapy, behavioral therapy, occlusal splints, physical modalities, and infiltrative therapy [9,10] in severe and persistent TMD cases refractory to conservative treatment, especially in the presence of significant structural joint abnormalities, advanced degenerative disease, ankylosis, trauma, or progressive functional impairment [11,12,13].

Ozone therapy has also been proposed as an adjunctive treatment for TMD; a systematic review reported improvements in pain reduction and maximal mouth opening in affected patients [14]. Botulinum toxin type A (BoNT-A) has gained a lot of attention for the treatment of chronic TMD, particularly the myogenous variant that entails discomfort and dysfunction of the temporalis, masseter and pterygoid muscles. It is a neurotoxin produced by Clostridium botulinum; its traditional mechanism of action involves blocking acetylcholine release at presynaptic nerve terminals, which results in temporary paralysis or decreased muscle activity [15].

The therapeutic effect of BoNT-A in TMD is not solely related to muscle relaxation, but also involves important direct analgesic properties. Pro-inflammatory mediators and neurotransmitters implicated in pain transmission, including glutamate, substance P, and CGRP (Calcitonin Gene-Related Peptide), seem to be inhibited by BoNT-A. Additionally, it may modulate the central nervous system through effects on GABAergic and opioid systems, reducing peripheral and central sensitization phenomena associated with chronic pain. Furthermore, some research suggests that BTX-A may lessen the functional load exerted on the temporomandibular joint, indirectly contributing to symptomatic improvement [16]. The most frequently reported adverse effects of botulinum toxin include muscle weakness, difficulty in chewing, and discomfort during mastication, which may, however, be transient and not consistently reported across studies, with some authors failing to observe clinically relevant functional impairment or a significant impact on quality of life. In addition, procedure-related effects have been described, including edema, ecchymosis, alterations in smiling, dysphagia, ptosis, and paresthesia [17]. Nevertheless, the overall incidence and clinical relevance of these adverse events remain heterogeneous in the literature, with several studies reporting minimal or no significant side effects following treatment. However, botulinum toxin has gained increasing clinical interest due to its reported effects in reducing pain, improving sleep quality and overall quality of life, decreasing bruxism episodes, lowering electromyographic activity of the masticatory muscles, and reducing morning mandibular stiffness. Several recent systematic reviews have evaluated the role of BoNT-A in TMDs; however, they differed substantially in design, eligibility criteria, included study types, and search periods [18]. Therefore, an updated synthesis focused specifically on recent randomized controlled trials remains clinically relevant.

The aim of this review is to evaluate the effectiveness of botulinum toxin in the rehabilitation management of TMDs with a specific focus on its comparison with conservative approaches, particularly saline solution injections, as reported in recent studies. In this context, increasing attention has been directed toward the potential equivalence between botulinum toxin and placebo or non-active interventions, raising important considerations regarding clinical benefit, cost-effectiveness, and resource allocation. If botulinum toxin does not demonstrate superior outcomes compared to saline solution, its routine clinical use may warrant reconsideration in favor of more conservative and economically sustainable treatment strategies.

## 2. Materials and Methods

This systematic review was conducted by searching the following databases: PubMed, Scopus and Google Scholar (Table 1). We searched for randomized controlled trials written in English and published between January 2020 and the date of the last search update. The final search was updated in August 2026.

This review was previously registered in the PROSPERO database for systematic review (CRD420261452763).

Two reviewers (A.S. and R.C. (Rossella Calciano)) independently screened those databases.

Studies were selected according to inclusion and exclusion criteria following the PRISMA statement [19]. Following removal of duplicate records, titles and abstracts were screened to identify relevant studies. Full-text articles were then assessed for eligibility, and studies fulfilling all predefined criteria were included in the final qualitative synthesis.

Reviewers (A.S. and R.C. (Rossella Calciano)) independently screened all papers for eligibility. Articles were considered eligible if they met the inclusion criteria according to PICO guidelines [20].

(P) Participants: adult patients diagnosed with persistent/refractory TMD, myofascial pain, and bruxism;

(I) Intervention: BoNT-A injections;

(C) Comparator: control groups with other treatments;

(O) Outcome measures: Pain reduction, functional improvement.

Only randomized controlled trials evaluating BoNT-A injections in adult patients with myogenous TMD, persistent or refractory masticatory myofascial pain, or bruxism were included. Eligible comparators were placebo, saline injection, occlusal splints, dry needling, manual therapy, pharmacological therapy, physical therapy, or other conservative/non-surgical treatments.

We excluded studies that were incomplete or not accessible, or for which data did not allow data extraction. Any disagreements between the reviewers during the study selection process were resolved through discussion until consensus was reached. If consensus could not be reached, a third reviewer (A.C.) was consulted and made the final decision.

The study selection and extraction process followed the PRISMA guidelines [19] (Figure 1).

The methodological quality of the included studies was assessed using the Cochrane Risk of Bias tool (RoB 2.0) through the RevMan Web software 11.7.0 (https://revman.cochrane.org/info) in 1 July 2026. This software provided the evaluation of seven key domains: random sequence generation, allocation concealment, blinding of participants and personnel, blinding of outcome assessment, incomplete outcome data, selective reporting, and other potential sources of bias. Each domain was judged as having a low, unclear, or high risk of bias, and results were visually summarized using a color-coded system: green for low risk, yellow for unclear risk, and red for high risk (Figure 2).

PRISMA 2020 for Abstracts Checklist, PRISMA Statement are available in Supplementary Material Section.

## 3. Results

We selected fifteen randomized controlled trials (RCTs), published between 2020 and 2025, evaluating the efficacy of botulinum toxin type A (BoNT-A) for the management of temporomandibular disorders (TMD) and bruxism (Table 2). Our review included 757 participants, of whom 119 were males and 638 were females. BoNT-A doses varied considerably, ranging from 50 U to 200 U, and follow-up periods ranged from 2 weeks to 9 months. Control interventions included saline placebo, occlusal splints, oral appliance therapy, lidocaine injections, dry needling, pharmacological therapy, and manual therapy. Clinical outcomes were assessed using a combination of pain tools, mandibular function, quality of life, and patients’ satisfaction.

The risk of bias assessment highlighted several methodological concerns among the included studies, particularly regarding the domain of blinding of outcome assessment. All studies presented some concerns in this domain, with the exception of De la Torre et al., Lennartsson et al., and Sitnikova et al. [3,21,23].

Furthermore, Angelo et al., Chisini et al., De la Torre et al., Lennartsson et al., Minston et al., Montes-Carmona et al., Reeve et al., Sahin et al., and Shin et al. were judged to be at high risk of bias in the incomplete outcome data domain [3,21,24,25,27,28,31,32,34].

Considerable variability was also observed across the included studies in terms of sample size, intervention protocols, outcome measures, and follow-up duration. These methodological differences, together with the limited sample sizes of several studies, may have affected the reliability and comparability of the findings. Overall, although the available evidence suggests a potential beneficial effect of botulinum toxin, the presence of relevant methodological limitations and substantial heterogeneity across studies precludes definitive conclusions regarding treatment efficacy. Further well-designed randomized controlled trials with larger sample sizes, standardized treatment protocols, adequate follow-up periods, and comprehensive outcome assessment and reporting are warranted to provide more robust evidence and enable a more precise estimation of the therapeutic effect.

### 3.1. Botulinum Toxin Versus Placebo

Twelve studies compared BoNT-A with saline placebo. Five of these RCTs reported no statistically significant superiority of BoNT-A over placebo. Sitnikova et al. observed improvements in myofascial pain symptoms following BoNT-A injection; however, these improvements were not significantly greater than those observed with saline placebo [21]. The same results were reported by Reeve et al., Ângelo et al., and Lennartsson et al., all of whom concluded that BoNT-A did not provide additional clinical benefit over placebo in patients with chronic myofascial TMD or chronic orofacial myalgia [23,25,28].

Six studies demonstrated significant benefits associated with BoNT-A treatment. Kim et al. reported a greater reduction in the number of tender points and an overall trend of pain improvement compared to the placebo [22]. Montes-Carmona et al. found both significantly greater reductions in pain intensity and improvements in mandibular function following BoNT-A treatment compared to saline injections [24]. Dela Torre Canales et al. reported significant improvements in mandibular range of motion and reductions in muscle tenderness compared with placebo, with no significant differences between low-, medium-, and high-dose BoNT-A protocols [30]. The same results were reported by De la Torre Canales et al. in patients with refractory masticatory myofascial pain [3]. Likewise, Montes-Carmona et al. included a lidocaine injection group and demonstrated superior pain reduction and functional improvement with BoNT-A compared with both saline and lidocaine [24]. Valli et al. observed that patients with localized myalgia experienced significantly greater pain relief than those presenting with widespread pain or systemic comorbidities [26]. Likewise, De la Torre Canales et al. demonstrated that BoNT-A significantly improved pain-related disability compared with saline placebo, although outcomes were comparable to those achieved with oral appliance therapy [29].

### 3.2. Botulinum Toxin Versus Conservative Therapies

Four RCTs compared BoNT-A with conservative treatment modalities. Karagozoglu and Ozcan compared BoNT-A with occlusal splints in patients with bruxism, reporting no significant differences between BoNT-A and occlusal splints, indicating equivalent therapeutic effectiveness [33]. Şahin et al. compared BoNT-A with dry needling, pharmacological treatment, and manual therapy in patients with bruxism-induced myalgia, reporting significant clinical improvement across all treatment groups without significant differences between interventions [32]. Two RCTs specifically investigated sleep bruxism. Shim et al. demonstrated that BoNT-A significantly reduced both the frequency and intensity of sleep bruxism episodes, as assessed by polysomnography and bruxism indices, showing superiority over placebo [31]. In contrast, Chisini et al. found that BoNT-A and occlusal splint therapy achieved similar clinical outcomes over a six-month follow-up period [34].

## 4. Discussion

The present systematic review evaluated the efficacy BoNT-A for the management of temporomandibular disorders (TMDs) and bruxism.

The findings of the present review should be interpreted in the context of recently published systematic reviews on BoNT-A for TMDs. Kharraz et al. included 23 studies published between January 2000 and June 2024 and evaluated BoNT-A in comparison with placebo and other therapies across several outcomes, including pain, maximum mouth opening, electromyographic activity, occlusal force, and adverse events. Compared with that broader review, the present review applied a narrower and more recent eligibility framework, focusing on randomized controlled trials published from 2020 onward and emphasizing comparisons with placebo, saline injections, and conservative/non-surgical treatments [35].

The paper includes 15 RCTs published between 2020 and 2025, trying to provide an updated of the available evidence. One potentially relevant study published in 2025 was identified but excluded at the full-text assessment stage because the full text could not be retrieved [36].

Although previous systematic review Saini RS et al. studied this theme there is a lack of a standardized protocol for BTX-A administration; this is one of the most important issue, because they vary in terms of patient populations, diagnostic criteria, botulinum toxin dosage, injected muscles, injection technique, follow-up duration, and outcome assessment [18,37]. BoNT-A may provide a reduction in pain relief and functional improvement in selected patients, particularly those with refractory myogenous TMD, localized myalgia, or severe bruxism. However, the evidence remains inconsistent regarding its superiority over placebo and conventional conservative therapies, highlighting the need for more careful patient selection and standardized treatment protocols. However, the results of the present review are consistent with the meta-analysis by Saini RS et al. concluded that BoNT-A did not demonstrate significant superiority over placebo in reducing pain intensity, improving maximum mouth opening, or decreasing bruxism episodes, despite positive results [18].

Another recent review De la Torre Canales et al. indicated that botulinum toxin may represent an effective therapeutic choice for patients who do not respond to conservative treatments, so patients experienced pain relief and benefit but the low evidence did not provide and support its routinary use in clinical practice. It reported a significant reduction in refractory myofascial pain and muscle tenderness compared with placebo and pain was measured with VAS. De la Torre Canales et al. included patients with refractory masticatory myofascial pain defined as those who had not responded to traditional treatments [3]. Similarly, Montes-Carmona et al. and Valli et al. have shown that botulinum toxin is more beneficial for patients with localized myofascial pain [24,26]. Our findings also suggest that treatment effectiveness may depend on patients type and pathology.

Valli et al. observed that individuals with localized pain benefited more from botulinum toxin therapy than those with systemic comorbidities or widespread pain, provided that botulinum toxin is not the only factor contributing to changes in VAS scores; other factors include the research population’s characteristics, the type of pain (localized versus general), the presence of central sensitization, and the comparator employed [26].

Studies enrolling patients with myogenous or refractory temporomandibular disorders generally reported better outcomes than those including heterogeneous TMD populations; this is consistent with Li et al., which demonstrated that BoNT-A was associated with significant pain reduction in patients with muscular TMD, particularly at 1- and 6-month follow-up suggesting that patients with muscular involvement may have more benefit from treatment. Although Li et al. also focused on randomized controlled trials and included a similar number of studies, the present review differs by incorporating more recent clinical trials published after their search period and by providing a broader clinical interpretation of comparator type, diagnostic heterogeneity, treatment dose, injection protocol, and follow-up duration. Therefore, the contribution of the present review is not only an updated study count, but also a more detailed interpretation of how patient phenotype and comparator choice may influence the apparent effectiveness of BoNT-A [38]. Furthermore, De la Torre Canales et al. concluded that although BoNT-A may reduce pain in patients with myogenous TMD, it is not superior to standard conservative treatments and should be reserved for refractory cases because of the potential risk of side effects. The authors in this umbrella review synthesized previous systematic reviews rather than primary RCTs. Therefore, its methodological scope differs from the present review, which directly examines recent randomized controlled trials. Nevertheless, both reviews support a cautious position: BoNT-A may be considered in selected patients with myogenous TMD or refractory symptoms, but it should not be recommended as a routine first-line treatment [3].

Shim et al. showed that BoNT-A was superior to a placebo in a disease like bruxism by demonstrating a significant decrease in masseter muscle activity and the frequency of bruxism episodes as measured by polysomnography (PSG) [31]. Montes-Carmona et al., Valli et al., De la Torre Canales et al. e Shim et al. showed that bruxism and several types of localized or refractory myofascial pain are associated with muscle hyperactivity. These findings highlighted that botulinum toxin aims to reduce masticatory muscle hyperactivity rather than to directly manage chronic pain [3,24,26,31].

Indeed, Lennartsson et al., Sitnikova et al., and Reeve et al. examined patients with chronic orofacial myalgia and found no additional therapeutic benefit of botulinum toxin when compared to saline or placebo [21,23,25].

An important consideration concerns the comparison with placebo, which did not demonstrate statistically significant differences between BoNT-A and saline injections. These findings suggest that part of the observed clinical improvement may be attributable to other factors, like the injection procedure itself, placebo response, and clinician’s experience. Nevertheless, given the multifactorial and biopsychosocial characteristics of TMDs, these aspects should be considered carefully [21,39].

Another important point is the comparison to other conservative treatments. This observation is supported by recent evidence, like the review by De la Torre Canales et al. concluded that although BoNT-A may reduce pain in patients with myogenous TMD, it does not provide superior outcomes compared with established conservative treatments and should therefore be reserved for refractory cases because of its potential adverse effect [30]. Likewise, Zhu et al. reported that BoNT-A provides pain relief comparable to conventional therapies, without clinically significant superiority in mandibular function or mouth opening. These findings highlight the need and the current recommendations that conservative therapies remain the first line of treatment [40].

One of the main challenges identified in this review is the marked methodological heterogeneity among the included studies. Variability was observed regarding BoNT-A dosage, number of injection sites, injected muscles, injection depth, use of electromyographic or ultrasound guidance, follow-up duration, diagnostic criteria, and outcome assessment tools. This lack of standardization weakens the comparison between studies and make difficult to find a standardized protocol. However, it is important to notice that there is a lack of uniformity in diagnostic criteria for TMDs, so this may explain the differences and the heterogeneity in reported treatment outcomes [2,41].

The dosage of botulinum toxin varied among the included studies. Sitnikova et al. noticed an improvement in pain symptoms as measured by the VAS with 50 U of BoNT-A to individuals with chronic myofascial pain; nevertheless, they were unable to show a clear advantage over saline [21]. When compared to a placebo, Shim et al. found a substantial decrease in the frequency and severity of sleep bruxism episodes using the same 50 U dose [17]. This apparent discrepancy may be explained by the fact that Shim et al. investigated patients with sleep bruxism, a condition in which reducing masseter muscle contractile activity represents the primary therapeutic mechanism of botulinum toxin. However, research on myofascial pain also includes central pain modulation processes, which could restrict BoNT-A’s total therapeutic efficacy [31].

Ângelo et al., Reeve et al., Lennartsson et al. and Minston et al. found no statistically significant differences between BoNT-A and placebo, despite administering a 100 U dose (intermediate doses), indicating that the clinical benefit may not completely dependent on the amount of botulinum toxin administered [23,25,27,28]. De la Torre Canales et al. demonstrated improvement in patients with refractory masticatory myofascial pain treated with 80 U of BoNT-A (intermediate dose) [3]. The studies of De la Torre Canales et al. showed that increasing the BoNT-A dose not always result in greater therapeutic efficacy. The different dosing regimens (80 U, 140 U, and 200 U) do not result a discernible variation in clinical outcome. Other factors, such as patient characteristics and the injection technique, may have a greater influence on clinical outcomes [29,30].

The considerable variability in follow-up duration, ranging from a few weeks to nine months, constituted another significant source of heterogeneity. Consequently, direct comparison of clinical outcomes was limited, and the long-term durability of the therapeutic effects of botulinum toxin could not be reliably established. Shim et al. evaluated patients for about 12 weeks (short follow-up times) and found that bruxism episodes and muscle activity had significantly decreased in the initial weeks following therapy [31]. Similarly, Montes-Carmona et al. and De la Torre Canales et al. reported improvements in myofascial pain and mandibular function in the initial months after injection of botulinum toxin. They demonstrated the mechanism of action of BoNT-A, whose pharmacological effect typically starts within a few days, peaks after a few weeks, and progressively diminishes when neuromuscular transmission is restored [3,24].

Lennartsson et al. [23]., Valli et al. [26] and Reeve et al. [25] suggested that botulinum toxin’s effectiveness may be transient. Although significant improvements were observed in the early stage, but decrease over the follow-up period, underscoring the necessity of carefully weighing the prospective need for recurrent injections against the length of therapeutic benefit]. All included studies aimed to improve the symptoms of temporomandibular disorders, but the outcome measures are different between the trials; therefore, the results of studies are heterogeneous.

Montes-Carmona et al. demonstrated that pain reduction was accompanied by a significant improvement in maximum interincisal opening (MIO) and mandibular function in patients treated with botulinum toxin [24]. Similarly, Shim et al. reported subjective results like pain reduction, but also improvements in objective parameters (polysomnographic findings and the bruxism index [31]). Chisini et al. and Karagozoglu et al. reported significant reduction in frequency of bruxism episodes and masseter muscle activity following BoNT-A treatment; however, they did not demonstrate superior clinical outcomes of botulinum toxin with occlusal splint therapy or other conservative treatments. These studies suggest that reduced muscle activity represents only one component of treatment efficacy and that the clinical benefit perceived by patients is also influenced by other functional and patient-related factors [33,34].

Therefore, it is important to underline that, despite the differences in diagnostic criteria, BoNT-A should be regarded as a personalized therapeutic option rather than a routine effective intervention. Careful patient selection, particularly identifying patients with refractory myogenous TMD who have failed conventional conservative management, may optimize treatment outcomes while minimizing unnecessary exposure to repeated injections [17,37].

### Limitations

Nevertheless, several limitations should be acknowledged. First, despite the use of a predefined search strategy and eligibility criteria, the possibility of selection and publication bias cannot be excluded. Second, the heterogeneity of the included studies in terms of study design, diagnostic criteria, outcome measures, and treatment protocols limited the comparability of the findings and precluded robust quantitative synthesis. In addition, the limited number of eligible studies may reduce the strength and generalizability of the overall conclusions. Finally, although the available evidence suggests a potential benefit of BoNT-A for some clinical outcomes in patients with TMDs, the methodological limitations of the available evidence and the heterogeneity across studies prevent definitive conclusions regarding its clinical effectiveness or its role as an optimal treatment option. Further high-quality systematic research and well-designed randomized controlled trials are warranted to strengthen the evidence base and better define the clinical role of BoNT-A in the management of TMDs.

## 5. Conclusions

The findings of this systematic review should be interpreted with caution. They suggest that botulinum BoNT-A may provide clinical benefits in selected patients with TMDs, particularly those with refractory myogenous TMD, localized myofascial pain, or severe bruxism characterized by masticatory muscle hyperactivity. Despite reductions in pain, bruxism activity and improvement in joint function, these benefits were not superior to placebo or to other conservative strategies like occlusal splints, oral appliance therapy, manual therapy, or pharmacological management.

Current evidence does not support the routine use of BoNT-A as a first-line treatment for TMDs because of the heterogeneity among the included studies. They differences in patient populations, diagnostic criteria, injection protocols, toxin dosage, treated muscles, follow-up duration, and outcome measures, with a limitation in the strength of the available evidence.

Based on the current literature, BoNT-A should be considered as a potential adjunctive or second-line therapeutic option for selected patients who do not respond adequately to conventional management. Clinical decision-making should remain individualized and consider the patient’s diagnosis, symptom profile, treatment history, and the potential benefits and risks associated with repeated injections. Future high-quality, adequately powered RCTs using standardized diagnostic criteria, injection protocols, clinically relevant and uniform outcome measures, and longer follow-up periods are needed to better define the indications, optimal dosage, duration of effect, and long-term safety of BoNT-A in the management of TMDs and bruxism.

## Figures and Tables

**Figure 1:**
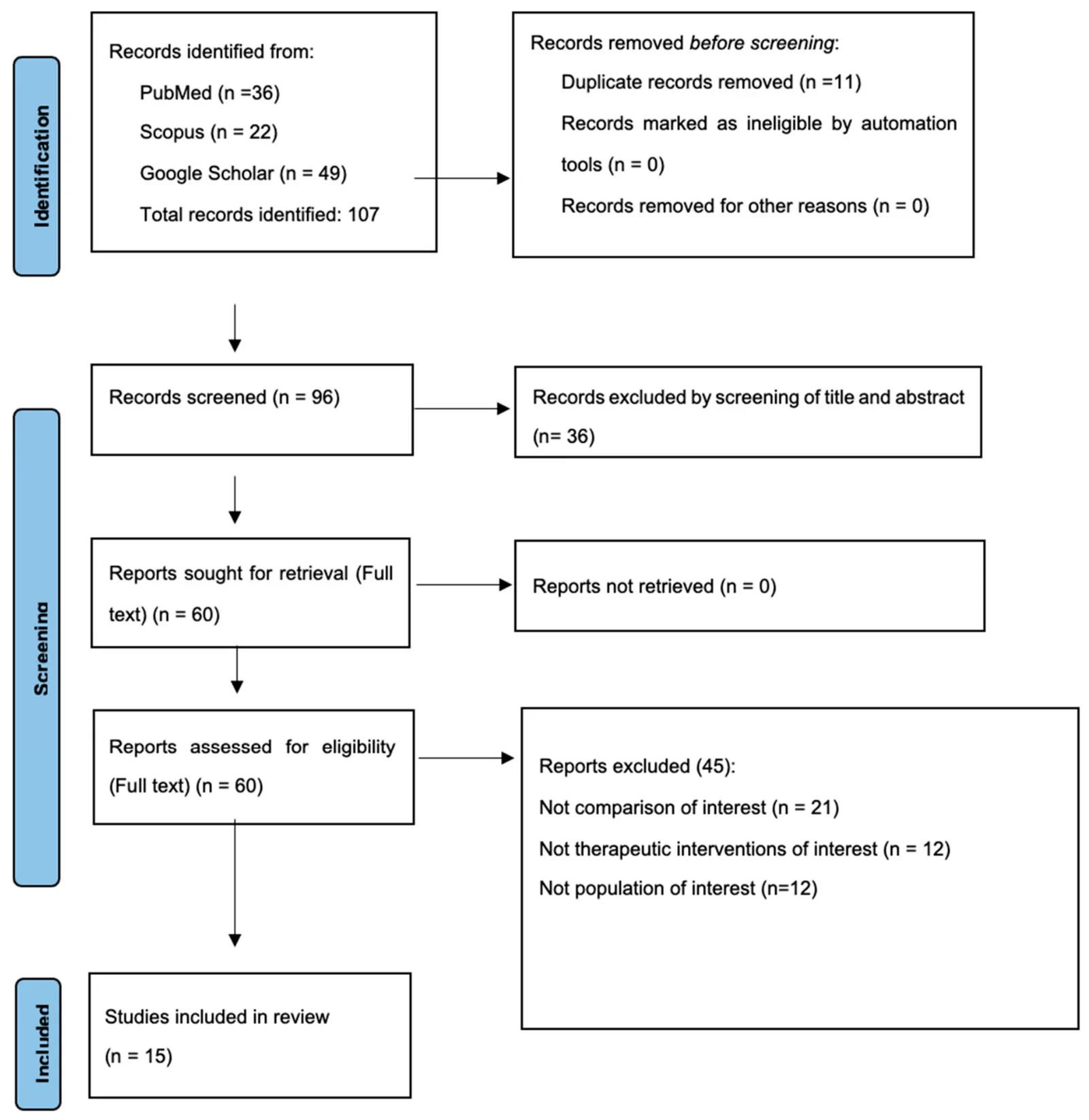
Study selection process assessed through PRISMA 2020 statement.

**Figure 2:**
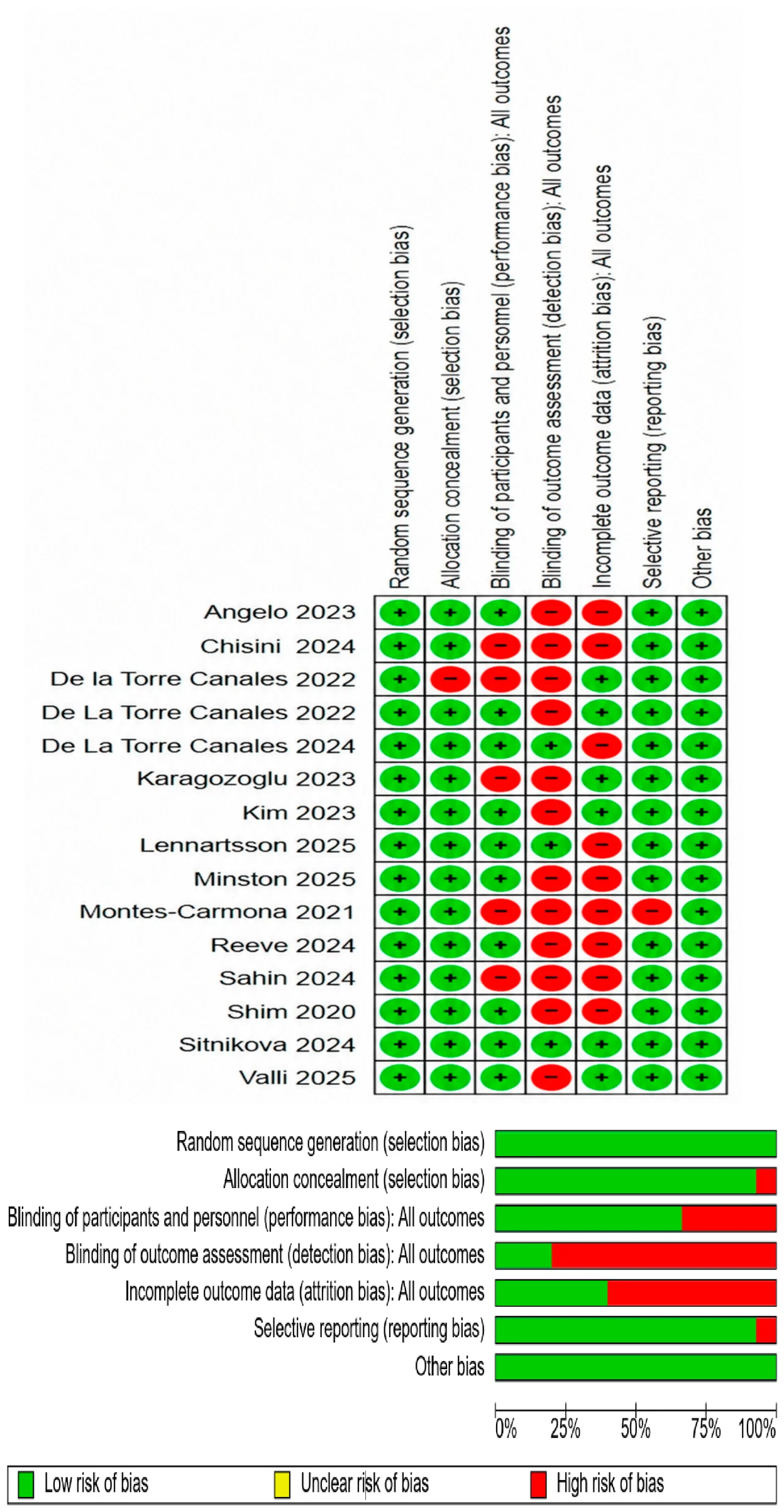
Risk of bias [3,21,22,23,24,25,26,27,28,29,30,31,32,33,34].

**Table 1:** Search strategy.

Database	Search Strategy	Filters Applied
PubMed	((Temporomandibular Joint Disorders OR temporomandibular joint OR TMJ OR temporomandibular disorder OR bruxism OR jaw pain) AND (Botulinum Toxins OR botulinum toxin OR botox OR onabotulinumtoxinA OR abobotulinumtoxinA OR incobotulinumtoxinA))	Last 10 years RCT
Scopus	TITLE-ABS-KEY (temporomandibular OR TMJ OR TMD) AND TITLE-ABS-KEY-AUTH (botulinum toxin OR botox) AND TITLE-ABS-KEY (placebo OR sham) AND TITLE-ABS-KEY (randomized OR RCT)	2020–2026 English
Google Scholar	(botulinum toxin type A or BTA) AND (masticatory myofascial pain syndrome OR TMD) AND (saline solution) AND (randomized controlled trial) NOT (click OR disk displacement) OR arthralgia OR osteoarthritis)	2020–2026 All documents (not only scientific articles)

**Table 2:** Characteristics of the studies.

Author (Year)	Study Design	Pathology	Study Group	Intervention Group I	Intervention Group II	Intervention Group III	Main Outcomes	Follow Up	Results
Sitnikova et al. (2024) [21]	RCT	TMD (Chronic Orofacial Myalgia)	Age: 38.2 ± 10.4 n: 57 M: 10 F: 47 BoNT-A 50 IU	Age: 38.2 ± 10.4 n: 57 M: 10 F: 47 Saline Solution			CPI, GCPS, NRS, Bite Force, EMG	(T0) baseline (T1) 2 weeks (T2) 11 weeks (T3) 16 weeks	Injections of 50 U of BoNT-A can improve myofascial pain symptoms, but its specific effect is not clearly superior to that of the placebo
Kim et al. (2023) [22]	RCT	TMD, (Orofacial Myalgia, Headache)	Age: 34.71 ± 9.73 n: 14 M: 1 F: 13 BoNT-A 150 IU	Age: 32.43 ± 10.23 n: 7 M: 1 F: 6 Saline Solution			OVAS, TPs, MMO, HF, HVAS	(T0) Baseline (T1) 4 weeks (T2) 8 weeks (T3) 12 weeks	BoNT/A significantly reduced the number of tender points compared with saline and showed improvements in orofacial pain and headache over time, suggesting partial clinical efficacy.
De la Torre Canales (2024) [3]	RCT	TMD (Refractory Masticatory Pain)	Age: 39.1 ± 11.9 n: 14 M: 0 F: 14 BoNT-A 80 IU	Age: 39.2 ± 10.9 n: 14 M: 0 F: 14 Saline Solution			VAS, QST, CPM, PHQ-9, GAD-7, PHQ-15	(T0) Baseline (T1) 1 month (T2) 6 months	BoNT-A showed significant clinical improvements compared with saline in patients with refractory masticatory myofascial pain.
Lennarts son et al. (2025) [23]	RCT	TMD (Chronic Orofacial Myalgia)	Age: 32.3 ± 11.0 n: 36 M: 2 F: h34 BoNT-A 100 IU	Age: 36.1 ± 11.5 n: 36 M: 5 F: 31 Saline Solution			VAS, GCPS, PGIC, Psychosocial scales, MMO	(T0) Baseline (T1) 1 month (T2) 3 months (T3) 4 months (T4) 6 months (T5) 9 months	The administration of two doses of 100 U of BTX-A did not provide additional clinical benefit over placebo for patients with chronic orofacial myalgia
Montes-Carmona (2021) [24]	RCT	TMD (Masticatory Pain)	Age: 42.4 ± 14.8 n: 20 M: 4 F: 16 BoNT-A 100– 150 IU	Age: 42.95 ± 15.4 n: 20 M: 3 F: 17 Saline Solution	Age: 45.40 ± 16.1 n: 20 M: 4 F: 16 Lidocaine 2%		VAS, MIO, Mandibular movements, TMJ Function questionnaire	(T0) baseline (T1) 1 weeks, (T2) 2 weeks (T3) 1 month (T4) 2 months (T5) 3 months (T6) 6 months	BoNT-A significantly reduced pain and improved mandibular function compared with saline solution and Lidocaine. The greatest benefit was observed in patients with localized myalgia and myofascial pain
Reeve et al. (2023) [25]	RCT	TMD (Myofascial Pain)	Age: 43.1 ± 14.8 n: 20 M: 2 F: 18 BoNT-A 100 IU	Age: 39.2 ± 14.0 n: 20 M: 2 F: 18 Saline Solution			VAS, MMO, OHIP-14	(T0) Baseline (T1) 1 month (T2) 3 months (T3) 6 months	BoNT-A is not superior to placebo for managing chronic masticatory myofascial pain.
Valli et al. (2025) [26]	RCT	TMD (Myofascial Pain)	Age: 41.7 n: 66 M: 8 F: 58 BoNT-A 50 IU	Age: 41.7 n: 66 M: 8 F: 58 Saline Solution			VAS, Pain drawings Likert scale	(T0) Baseline (T1) 1 month (T2) 4 months	Patients presenting with localized pain reported significantly greater relief compared to those with widespread pain or systemic comorbidities.
Minston et al. (2025) [27]	RCT	TMD (Myalgia)	Age: 36.8 ± 5.6 n: 23 M: 2 F: 21 BoNT-A 100 IU	Age: 36.8 ± 5.6 n: 22 M: 4 F: 18 Oral Appliance and Saline Solution			GCPS, CPI, SCL-DEP, SCL-SOM	T0: baseline T1: 6 months	BoNT-A outcomes were not significantly better than saline placebo and comparable to oral appliance therapy.
Ângelo et al. (2023) [28]	RCT	TMD (Myalgia)	Age: 37.3 ± 10.7 n: 23 M: 2 F: 21 BoNT-A 100 IU	Age: 33.8 ± 10.8 n: 22 M: 4 F: 18 Saline Solution			VAS	T0: baseline T1: 2 weeks T2: 1 month T3: 2 months	No statistically significant difference between the two groups.
De La Torre Canales et al. (2022) [29]	RCT	TMD (Myalgia)	Age 36.8 ± 5.6 n: 60 M: 0 F: 60 BoNT-A Low dose: 80 IU Medium dose: 140 IU High dose: 200 IU	Mean age 36.8 ± 5.6 n: 20 M: 0 F: 20 Saline Solution (SS) n = 20			PFMOMUO, MAO, Muscle tenderness to palpation (RDC/TM D Axis I).	T0 (baseline), T1 (28 days), T2 (180 days).	BoNT-A significantly improved mandibular range of motion and reduced muscle tenderness compared with placebo. Improvements occurred regardless of toxin dose.
De la Torre Canales et al. 2022 [30]	RCT	TMD (Myalgia)	Age: 36.8 ± 5.6 n: 60 M: 0 F: 60 BoNT-A Low Dose 60 IU BoNT-A Medium Dose BoNT-A High Dose	Age: 36.8 ± 5.6 n: 20 M: 0 F: 40 Oral Appliance	Age: 36.8 ± 5.6 n: 20 M: 0 F: 20 Saline Solution		GCPS, CPI, SCL-DEP, SCL-SOM	T0:baseline T1: 6 months	BoNT-A outcomes were significantly better than saline placebo and comparable to oral appliance therapy. No significant differences among the different BoNT-A doses.
Shim et al. (2020) [31]	RCT	Sleep Bruxism	Age: 27.7 ± 5.9 n: 13 M: 6 F: 7 BoNT-A 50 IU	Age: 28.7 ± 7.1 n: 10 M: 4 F: 6 Saline Solution			PSG, Bruxism index, VAS	(T0) Baseline (T1) 4 weeks (T2) 8 weeks (T3) 12 weeks	Injections of BoNT-A effectively reduce both the frequency and the intensity of sleep bruxism, showing a clear superiority over the placebo
Şahin et al. (2024) [32]	RCT	Bruxism-induced Myalgia	Age: 29.15 ± 8.04 n: 20 M: 1 F: 19 BoNT-A 50 IU	Age 30.60 ± 9.38 n: 20 M: 10 F: 10 Dry Needling	Age 31.95 ± 9.21 n: 20 M: 9 F: 11 Pharmacotherapy	Age 28.50 ± 6.84 n: 20 M: 10 F: 10 Manual Therapy	VAS, MMO, OHIP-14	(T0) Baseline (T1) 2 weeks (T2) 4 weeks (T3) 12 weeks	Clinical outcomes improved significantly and equally across all four therapeutic arms.
Karagozoglu I. Ozcan M. (2023) [33]	RCT	Bruxism	Age: 35.2 ±10.1 n: 20 M: 7 F: 13 BoNT-A 60 IU	Age: 33.8 ± 9.6 n: 20 M: 8 F: 12 Occlusal Splint			GCF Biomarkers (Cortisol, DHEA, TNF-α)	(T0) Baseline (T1) 3 months	Both botulinum toxin and occlusal splints are equally effective
Chisini et al. (2024) [34]	RCT	Sleep Bruxism	Age: 33.4 ± 10.9 n: 30 M: 9 F: 21 BoNT-A 60 IU	Age: 32.8 ± 10.8 n: 30 M: 9 F: 21 Occlusal Splint (OS)			GCPS, OHIP-14, MMO, Mandibular movements	(T0) Baseline (T1) 3 months (T2) 6 months	Both treatments are equally effective. Occlusal splints showed slight functional advantages

Abbreviations: BoNT-A, Botulinum toxin type A; CPI, Characteristic Pain Intensity; CPM, Conditioned Pain Modulation; DHEA, Dehydroepiandrosterone; EMG, Electromyography; F, Female; GAD-7, Generalized Anxiety Disorder 7-item Scale; GCF, Gingival Crevicular Fluid; GCPS, Graded Chronic Pain Scale; HF, Headache Frequency; HVAS, Headache Visual Analog Scale; M, Male; MAO, Maximum Assisted Opening; MIO, Maximum Interincisal Opening; MMO, Maximum Mouth Opening; MUO, Maximum Unassisted Opening; NRS, Numeric Rating Scale; OHIP-14, Oral Health Impact Profile–14; OS, Occlusal Splint; OVAS, Orofacial Visual Analog Scale; PFMO, Pain-Free Mouth Opening; PGIC, Patient Global Impression of Change; PHQ-9, Patient Health Questionnaire-9; PHQ-15, Patient Health Questionnaire-15; PSG, Polysomnography; QST, Quantitative Sensory Testing; RCT, Randomized Controlled Trial; RDC/TMD, Research Diagnostic Criteria for Temporomandibular Disorders; SCL-DEP, Symptom Checklist–Depression Subscale; SCL-SOM, Symptom Checklist–Somatization Subscale; SS, Saline Solution; TMJ, Temporomandibular Joint; TMD, Temporomandibular Disorder(s); TNF-α, Tumor Necrosis Factor Alpha; TPs, Tender Points; U, Units; VAS, Visual Analog Scale.

## Data Availability

No new data were created or analyzed in this study.

## Supplementary Materials

The following supporting information can be downloaded at: https://www.mdpi.com/article/10.3390/toxins18100417/s1. File S1: PRISMA 2020 for Abstracts Checklist; File S2: PRISMA 2020 Checklist.
